# Preventing enzymatic degradation of demineralized dentin collagen using a natural crosslinker

**DOI:** 10.1007/s00784-025-06719-1

**Published:** 2025-12-27

**Authors:** Anton Schestakow, Iris Xiaoxue Yin, Chun Hung Chu

**Affiliations:** Faculty of Dentistry, Prince Philip Dental Hospital, The University of Hong Kong, 34 Hospital Road, Hong Kong, China

**Keywords:** Aldehydes, Collagen, Cross-linking reagents, Dentin, Hydrolases, Tooth demineralization

## Abstract

**Objectives:**

To evaluate effectiveness of cinnamaldehyde, vanillin and dialdehyde starch in stabilizing collagen in demineralized dentin against enzymatic degradation.

**Materials and methods:**

Demineralized dentin collagen films were prepared from human teeth and treated for 3 min with 4% cinnamaldehyde, 4% vanillin or 4% dialdehyde starch. Deionized water and 4% glutaraldehyde served as negative and positive control. Crosslinker-collagen interaction was analysed using Fourier transform infrared spectroscopy (FTIR) (3 samples per group). After enzymatic degradation with collagenase type II, degradation was assessed via hydroxyproline assay (16 samples per group). Collagen network ultrastructure was examined using transmission electron microscopy (TEM) (2 samples per group). Data were analysed using the Kruskal-Wallis test and Dunn’s multiple comparison (*p* = 0.05).

**Results:**

FTIR confirmed that cinnamaldehyde, vanillin and dialdehyde starch did not disrupt the collagen triple-helix. Hydroxyproline release (µg) from dentin treated with water, vanillin, cinnamaldehyde, dialdehyde starch and glutaraldehyde were 15.5 ± 6.4, 13.6 ± 8.0, 11.1 ± 6.7, 6.1 ± 4.3 and 0.9 ± 0.8 (water, vanillin, cinnamaldehyde > dialdehyde starch, glutaraldehyde; *p* < 0.05). TEM revealed intact collagen fibrils in dentin treated with glutaraldehyde and dialdehyde starch, but not the dentin treated with water, vanillin and cinnamaldehyde.

**Conclusions:**

Among the three naturally derived aldehydes, this laboratory study showed that dialdehyde starch could be a promising cross-linking agent to stabilize demineralized dentin collagen.

**Clinical relevance:**

Incorporating natural crosslinkers such as dialdehyde starch into preventive strategies may improve the preservation of demineralized dentin by stabilizing the collagen matrix.

**Supplementary Information:**

The online version contains supplementary material available at 10.1007/s00784-025-06719-1.

## Introduction

Advancements in dental care and increased life expectancy have resulted in more individuals retaining their natural teeth into older age [1]. While this is a positive development, it also presents new challenges to oral health, particularly due to prolonged exposure of teeth to mechanical and chemical challenges. Among these, erosion has emerged as a growing concern, especially in populations where changing lifestyles and the widespread consumption of acidic foods and beverages may contribute to its increasing prevalence [2, 3].

Dental erosion is defined as the loss of dental hard tissue caused by acids or chelating agents in the absence of bacterial involvement [4]. In its early stages, erosion is often asymptomatic and therefore difficult to detect. By the time clinical signs become apparent, the dentin is frequently exposed [5, 6]. Demineralization of dentin leaves behind a collagen network that, while initially protective, becomes susceptible to enzymatic degradation by endogenous collagenases [7, 8]. This enzymatic degradation compromises the collagen’s ability to act as a protective barrier and as a scaffold for remineralization [9, 10], accelerating tooth wear and increasing the risk of hypersensitivity [11].

Beyond behavioural and preventive strategies, chemical cross-linking may help preserve demineralized dentin. Although extensively studied in restorative dentistry, its potential role in erosion lies in increasing the enzymatic resistance of demineralized dentin while maintaining its function in remineralization. However, these assumptions are primarily based on in vitro studies, and clinical evidence remains scarce [12, 13]. Among synthetic cross-linkers, glutaraldehyde has high efficacy in stabilizing collagen by forming covalent bonds with lysine residues through Schiff base reactions [14, 15]. However, glutaraldehyde is an irritant, and exposure was associated with various adverse health effects [16]. In dentistry, it is often used in light-curing adhesives. While in vitro studies demonstrate its cytotoxicity [13, 17], clinical data on long-term use and potential adverse effects remain limited [18].

Nevertheless, preventive agents intended for daily oral hygiene may allow for more frequent application and could offer potential benefits in the prevention and treatment of dental erosion [19]. In search of safer alternatives, naturally derived aldehydes have attracted interest due to their generally favourable biocompatibility, low toxicity, and cost-effectiveness [20]. Among these, cinnamaldehyde, an essential oil extracted from cinnamon, and vanillin, a phenolic aldehyde from vanilla pods, are capable of forming covalent bonds with collagen and have shown potential in enhancing the mechanical properties of biopolymers such as collagen [21, 22]. Another promising compound is dialdehyde starch, obtained by oxidizing plant-derived starch. It contains multiple aldehyde groups, enabling extensive cross-linking, and has been shown to improve collagen resistance in non-dental tissues [23, 24]. Despite these findings, the potential of natural aldehyde-based cross-linkers to stabilize demineralized dentin collagen, a substrate with distinct structural and biochemical characteristics, has not been comprehensively investigated. Most existing studies have focused on synthetic agents or examined natural cross-linkers in non-dental applications or with different outcomes, such as mechanical properties.

Therefore, this study aims to address this gap by investigating the effects of cinnamaldehyde, vanillin and dialdehyde starch on the stabilization of demineralized dentin collagen against enzymatic degradation. Using FTIR, hydroxyproline assay, and TEM, the structural integrity and degradation resistance of treated demineralized dentin were assessed. Glutaraldehyde was included as a positive control, while water served as a negative control. The findings seek to identify biocompatible alternatives to glutaraldehyde that could contribute to novel strategies for the prevention and management of erosion.

## Methods

### Sample preparation

The coronal third of intact human molars, stored in 0.1% thymol at 4 °C, was sectioned into 5 × 5 mm dentin slices using a custom-made saw. The dimensions were verified with a digital micrometer. Slices were demineralized in 12.5% EDTA in PBS (pH 7.2) for 6 d at room temperature (RT) under agitation. Complete demineralization was confirmed by microCT (SkyScan 1172; SkyScan, Antwerp, Belgium). Non-collagenous phosphoproteins were extracted using 4 M guanidine hydrochloride in a buffer containing 0.5 M NaCl and 50 mM Tris-HCl (pH 7.4) for 24 h to minimize uncontrolled autohydrolysis of demineralized dentin. Following an ascending sucrose infiltration series, slices were embedded in optimal cutting temperature (OCT) compound and sectioned into 40 μm-thick collagen films using a cryostat microtome (CM1850, Leica Cryostat CMS, Wetzlar, Germany). Samples were stored in PBS at −20 °C until use [25, 26].

### Treatment

Collagen films were washed with PBS 3× for 10 min each. Excess liquid was blotted off, and the moist films were treated with 200 µL of one of the following solutions for 3 min, as previously described for dentin collagen [14]: distilled water (negative control); 4% (v/v) cinnamaldehyde in 100% ethanol; 4% (w/v) vanillin in 100% ethanol; 4% (w/v) dialdehyde starch in water; or 4% (w/w) glutaraldehyde in water (positive control) (Table 1). To prepare dialdehyde starch, 40 mg of the powder was dissolved in water to a final volume of 1 mL by heating at ~ 80 °C with stirring (500 rpm) for 45 min, followed by cooling to RT. All solutions were freshly prepared. After treatment, the films were washed with PBS 3× for 10 min each.

**Table 1:**
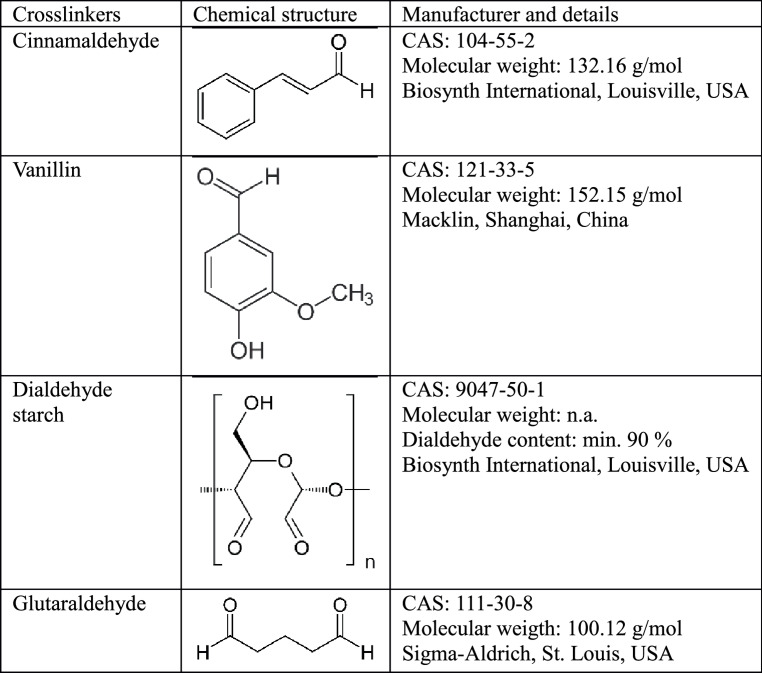
Crosslinkers used in this study

### FTIR

Structural changes in collagen films (*n* = 3 per group) were analysed using a Fourier-transform infrared (FTIR) spectrometer (Vortex 70, Bruker, USA) equipped with an MTC-detector and operated in attenuated total reflectance (ATR) mode. Prior to analysis, samples were thoroughly rinsed in distilled water (3 × 10 min) and subsequently freeze-dried. Spectra were acquired at a resolution of 1 cm⁻¹ over a wavenumber range of 4000–400 cm⁻¹, with 32 scans averaged per sample. Measurements were conducted in dry air at RT. Data analysis was performed using OriginPro 2021 (OriginLab, Northampton, USA), and spectra were normalized to the amide I band [26, 27].

### Degradation

Enzymatic degradation of collagen films (*n* = 16 per group) was performed using 100 µL of collagenase type II from Clostridium histolyticum (1 mg/mL; 125 U/mg; MSE Supplies, Tucson, USA) in 50 mM HEPES buffer (pH 7.4) containing 5 mM CaCl₂ and 0.002% NaN₃. Degradation was carried out for 20 min at 37 °C. The degradation solution was collected for hydroxyproline assay, and collagen films (*n* = 2 per group) were processed for TEM [27, 28].

### Hydroxyproline assay

The degradation solution (*n* = 16 per group) was gently mixed with 100 µL of 4 N NaOH and hydrolyzed at 120 °C for 1 h. After cooling to RT, 100 µL of 4 N HCl was added to neutralize the pH. Subsequently, 625 µL of chloramine-T reagent, containing 0.056 M chloramine-T in acetate-citrate buffer (pH 6.5) with 10% (v/v) propanol, was added to 300 µL of the hydrolysate, mixed gently, and incubated for 30 min at RT in the dark under agitation to allow oxidation. Then, 625 µL of freshly prepared Ehrlich’s reagent, containing 1 M Ehrlich’s aldehyde in 30% (v/v) HCl and 70% (v/v) propanol, was added, mixed gently, and the chromophore was developed by incubation at 65 °C for 20 min. Absorbance was measured at 550 nm using a microplate reader (SpectraMax M2, Molecular Devices, San Jose, USA) [29, 30]. Hydroxyproline concentration was determined using a standard curve from freshly prepared 2–20 µg hydroxyproline standards (trans-4-hydroxy-L-proline, analytical standard, Sigma-Aldrich, St. Louis, USA) for each assay.

### TEM

Collagen films (*n* = 2 per group) were fixed for 1 h with 1% glutaraldehyde and 1% paraformaldehyde in 0.1 M cacodylate buffer (pH 7.4), followed by 5 × 10 min washes in the same buffer. Post-fixation was performed with 2% osmium tetroxide in 0.1 M cacodylate buffer for 1 h in the dark. Samples were then washed 3 × 5 min with distilled water, dehydrated through an ethanol and acetone series, and embedded in epoxy resin (TAAB 812 Resin Kit, TAAB Laboratory Equipment, Berks, UK). Ultrathin Sect. (70 nm) were prepared using an ultramicrotome equipped with a diamond knife, mounted on copper grids, and stained with UranyLess (30 min) followed by lead citrate (20 min). The ultrastructure of the collagen network was examined descriptively at 5000× magnification using a Tecnai G2 20 scanning TEM (FEI Company, Hillsboro, USA). Untreated and undegraded samples (*n* = 2) served as additional controls [28, 31].

### Statistics

The sample size for the hydroxyproline assay was determined using G*Power software [32], based on an assumed large effect size (f = 0.4), a statistical power of 80%, and an α-error probability of 0.05. This resulted in a total of 80 collagen films (*n* = 16 per group). Statistical analysis was performed using GraphPad Prism 9 (GraphPad Software, San Diego, CA, USA). Normality of the hydroxyproline data was assessed using the Shapiro–Wilk test, which indicated that the data were not normally distributed. Differences between the negative control and other treatment groups were evaluated using the Kruskal–Wallis test, followed by Dunn’s multiple comparison test, with statistical significance set at *p* = 0.05.

## Results

### FTIR

**Fig. 1:**
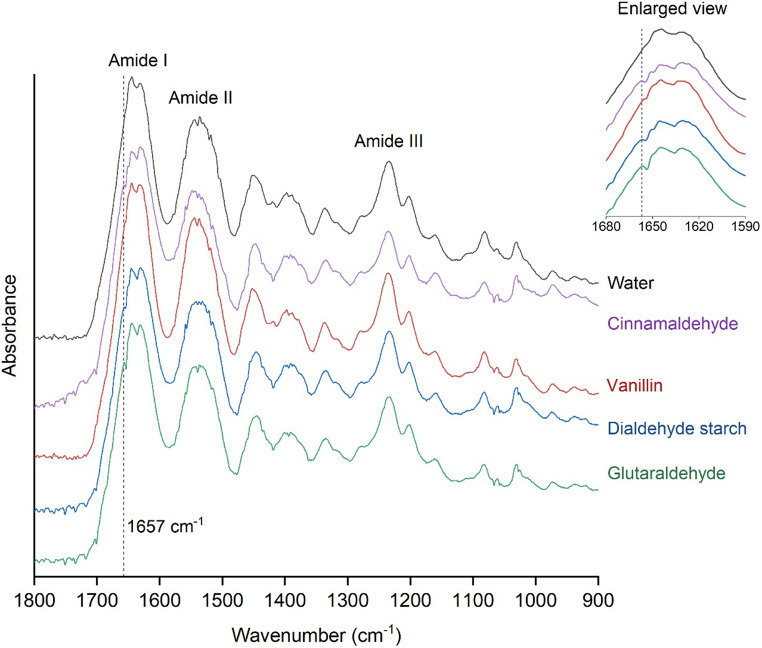
Fingerprint region of FTIR spectra of demineralized dentin films treated with water or cross-linkers

Characteristic FTIR bands were consistently identified in dentin films from both water- and cross-linker-treated groups, with no differences in band positions (Fig. 1; Table 2). The amide A band at 3295 cm⁻¹ and amide B band at 3075 cm⁻¹ correspond to N–H stretching coupled with hydrogen bonding and O–H stretching, respectively. The amide I band at 1630 cm⁻¹ reflects C = O stretching, while the amide II band at 1536 cm⁻¹ is attributed to N–H bending and C–N stretching. The amide III band, observed at 1236 cm⁻¹, arises from C–N stretching and N–H bending of amide linkages, as well as –CH₂ wagging vibrations of the glycine backbone and proline side chains [26, 33–35]. However, compared to the negative control, the band at 1657 cm⁻¹ was more intense in all cross-linker-treated groups, which may indicate the increase of C = N bonds as a result of the Schiff base reaction [36, 37]. The integrity of the collagen triple helix, indicated by the A₁₂₃₆/A₁₄₅₀ absorption ratio, remained preserved following treatment with cinnamaldehyde (0.989), vanillin (0.989), dialdehyde starch (0.988), and glutaraldehyde (0.992), compared to the negative control (0.992) [35].

**Table 2:** Band positions (cm^− 1^) in FTIR spectra of demineralized dentin films treated with water or cross-linkers

	Amide A	Amide B	Amide I	Amide II	Amide III
Water	3295	3074	1630	1536	1236
Cinnamaldehyde	3295	3075	1631	1537	1236
Vanillin	3297	3074	1630	1537	1237
Dialdehyde starch	3295	3074	1631	1537	1236
Glutaraldehyde	3295	3075	1631	1536	1236

### Hydroxyproline assay

**Fig. 2:**
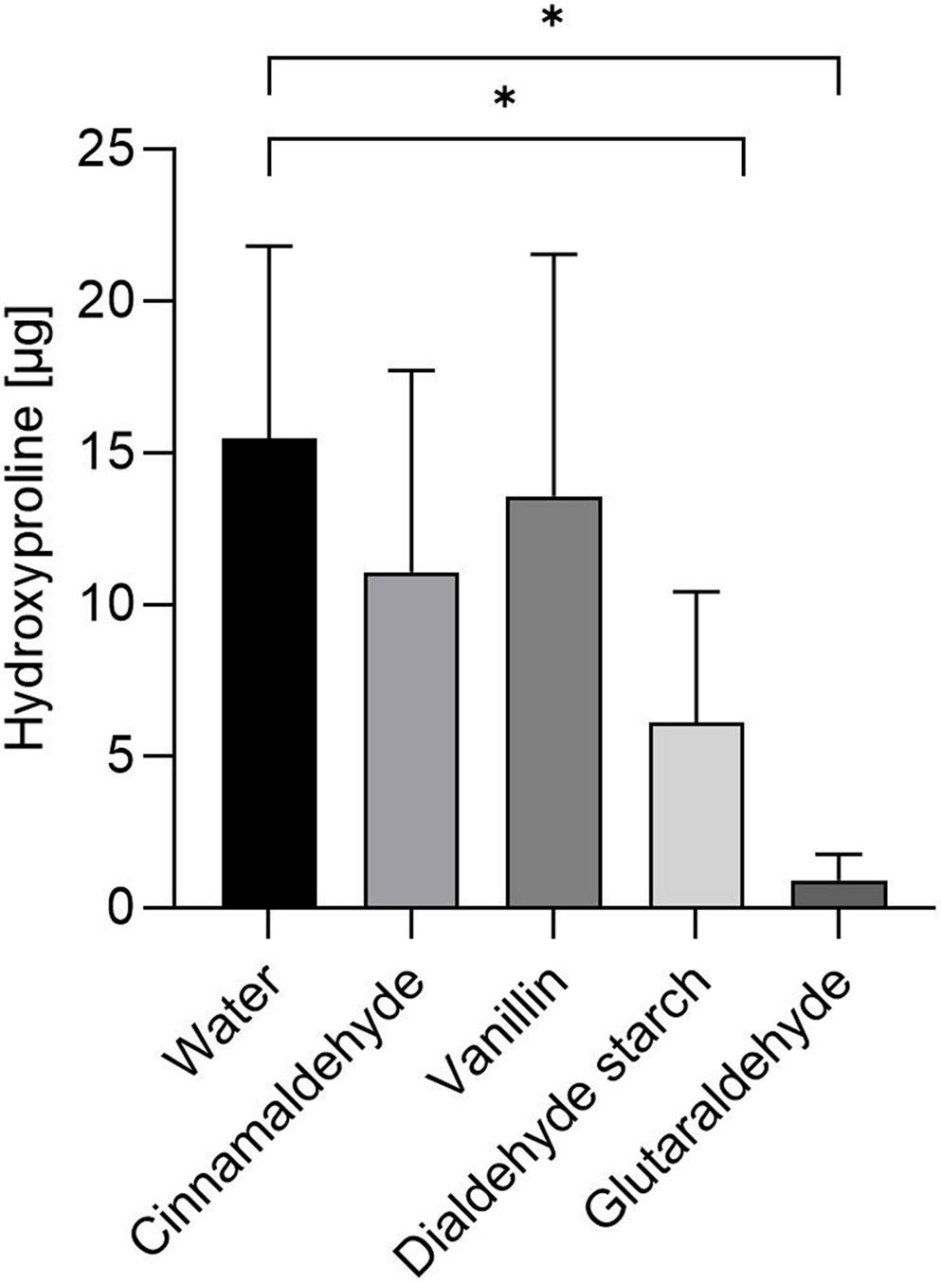
Hydroxyproline amount (mean ± SD) in the degradation solution. Significant differences compared to the water-treated control group are indicated by an asterisk (*p* < 0.05)

A hydroxyproline release of 15.5 ± 6.4 µg was measured in the degradation solution of dentin treated with water alone (Fig. 2). This amount did not differ significantly from samples pretreated with cinnamaldehyde (11.1 ± 6.7 µg) or vanillin (13.6 ± 8.0 µg). In contrast, pretreatment with dialdehyde starch (6.1 ± 4.3 µg) and glutaraldehyde (0.9 ± 0.8 µg) resulted in a significant reduction in degradation (*p* < 0.05).

### TEM

**Fig. 3:**
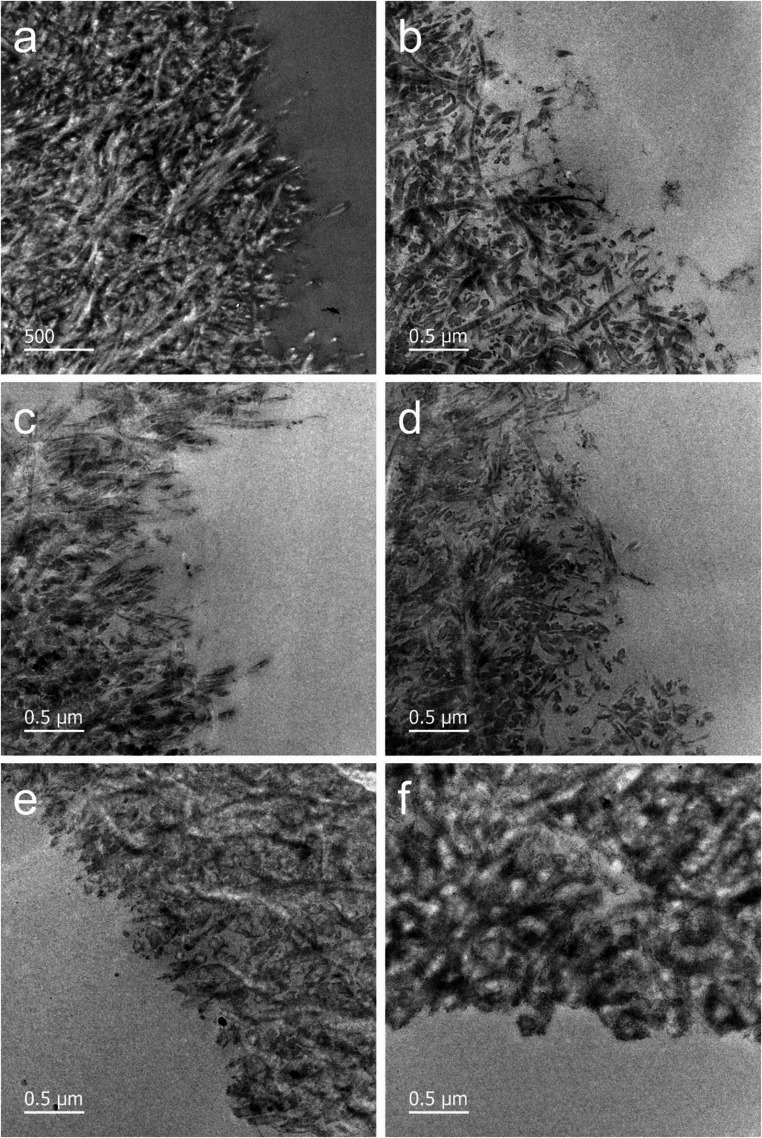
Representative TEM images of dentin films: untreated before degradation (**a**), and after degradation following pretreatment with water (**b**), cinnamaldehyde (**c**), vanillin (**d**), dialdehyde starch (**e**), and glutaraldehyde (**f**)

The TEM images show the ultrastructure of the demineralized dentin films with the exposed collagen network (Fig. 3). The collagen fibrils appear either in transverse orientation as round structures or in longitudinal orientation, displaying the characteristic banding pattern. Before degradation, the collagen network is dense and organized (Fig. 3a). After degradation, samples pretreated with water exhibited a disrupted collagen network, characterized by enlarged interfibrillar spaces and the presence of degraded collagen fragments (Fig. 3b). A similar pattern was observed in samples pretreated with cinnamaldehyde and vanillin (Fig. 3c, d). In contrast, samples treated with dialdehyde starch showed only slightly enlarged interfibrillar spaces (Fig. 3e), while those pretreated with glutaraldehyde maintained a dense collagen network without visible collagen degradation (Fig. 3f).

## Discussion

Maintaining the structural integrity of demineralized dentin is beneficial for both preventive strategies and the long-term stability of adhesive interfaces. In this study, dialdehyde starch demonstrated effective collagen stabilization against enzymatic degradation, outperforming other naturally derived cross-linkers. To evaluate this, thin, demineralized dentin films were employed. These films serve as a reliable model for quantifying collagen degradation and allow for complete agent penetration and homogeneous distribution [26, 27]. Moreover, under clinical scenarios such as erosion or intentional acid etching, demineralization typically affects only the superficial micrometer range [31, 38], mitigating the use of thick samples.

Treatment with dialdehyde starch or glutaraldehyde significantly reduced enzymatic degradation, as indicated by lower hydroxyproline levels. These results were supported by qualitative observations, in which films treated with either agent retained their network integrity after collagenase exposure. In contrast, films treated with water or other cross-linkers exhibited pronounced degradation. The aromatic aldehydes cinnamaldehyde and vanillin, which each possess only one reactive aldehyde group, showed limited protective effects. Their inability to form extensive cross-links likely accounts for their reduced efficacy. Although aromatic aldehydes may also engage in non-covalent hydrophobic interactions with proteins, these interactions are inherently weaker than covalent bonds and may be further hindered by their hydrophobic nature, which required dissolution in ethanol, a solvent known to influence the structure and stability of hydrated collagen [39]. Although FTIR spectra suggested preservation of the collagen triple helix, likely due to the short ethanol exposure and the highly organized network of dentin collagen. Ethanol may even enhance the interaction between lipophilic agents and collagen by mobilizing nonpolar amino acid residues [40]. While both aromatic aldehydes have shown benefits in other biopolymer systems [21, 22], their limited cross-linking potential and insufficient blocking of enzymatic cleavage sites likely limit their utility in protecting demineralized dentin.

In contrast, glutaraldehyde and dialdehyde starch contain multiple aldehyde groups, enabling extensive covalent cross-linking via Schiff base reactions, as supported by the intensified FTIR band at 1657 cm⁻¹ [14, 23, 36, 41]. However, no other distinct spectral changes were observed, suggesting that the collagen structure remained largely intact. This may be attributed to the low concentration and short exposure time used. Compared to the two aromatic aldehydes, glutaraldehyde and dialdehyde starch are also water-soluble, reducing the need for potentially irritating or cytotoxic solvents [42]. While glutaraldehyde is highly effective, its clinical use is limited due to its cytotoxicity [13, 17]. Dialdehyde starch offers a safer alternative with lower toxicity [22], though with reduced efficacy in collagen stabilization.

Differences in substrate type may influence cross-linking outcomes. Jayakumar et al. (2015) reported a 90% increase in collagenase resistance in reconstituted collagen films treated with a 1:1 dialdehyde starch solution for 24 h [23], while Wang et al. (2015) observed only a 40% increase in resistance in decellularized porcine aortic tissue treated with 5% dialdehyde starch for 3 d [41]. Despite shorter treatment times, our results in native dentin were comparable to Wang et al. (2015), suggesting that prolonged exposure may not enhance outcomes. The reduced efficacy observed in both Wang et al. (2015) and the current study, relative to Jayakumar et al. (2015), may be attributed to differences in the collagen substrate. Reconstituted collagen membranes, as used in Jayakumar et al. (2015), have minimal intrinsic cross-linking and are therefore more susceptible to chemical stabilization [23, 41]. In contrast, native collagen in soft and especially hard tissue is already extensively cross-linked [43, 44], which may limit the effectiveness of additional cross-linking agents. This is particularly relevant in dentin, which, unlike bone, does not undergo turnover. Cross-linking density in dentin varies by tooth type and location, increasing from incisors to molars, crowns to roots, and pulp to enamel, reflecting functional adaptations [45–47]. As this study focused on coronal dentin of molars, it remains uncertain whether cross-linking agents like dialdehyde starch are equally effective on more densely cross-linked cervical dentin or on less cross-linked teeth such as incisors.

Beyond stabilization, cross-linking agents may also promote remineralization. Glutaraldehyde has been shown to accelerate remineralization in the presence of amorphous calcium phosphate. This effect might result from intermediates formed with amino acid residues and residual aldehyde groups, which may act as additional nucleation sites [48]. Whether dialdehyde starch exhibits similar effects merit further investigation.

Nevertheless, the effectiveness of dialdehyde starch compared to other natural cross-linkers such as polyphenols, hesperidin, and riboflavin remains unknown [13], and its performance under oral cavity conditions, including the presence of saliva, the pellicle, and microorganisms, also needs to be clarified. Although microbial and salivary amylases are known to hydrolyse starch, the biodegradability of dialdehyde starch in the oral environment has not been specifically studied. However, findings from other fields suggest that dialdehyde starch exhibits greater resistance to enzymatic degradation than non-oxidized starch [49, 50]. Additionally, the pellicle may act as a physical barrier, limiting the diffusion of cross-linking agents into demineralized dentin [51]. Recent studies further indicate that salivary proteins can infiltrate the collagen network during demineralization, potentially restricting the accessibility of high molecular weight compounds such as dialdehyde starch and impeding their interaction with the collagen [52, 53]. Lastly, only a single concentration was evaluated in this study. A 4% concentration was used as an initial screening level, falling within the commonly reported effective range and enabling direct comparison between compounds [22]. Although lower concentrations have shown efficacy, these results are primarily based on reconstituted collagen [21, 23], or other biopolymers [22]. To the authors’ knowledge, no direct comparison exists between cross-linked reconstituted collagen and dentin in terms of resistance to degradation [48], supporting the use of a higher concentration in this study. Future research will systematically evaluate a broader range of concentrations to determine optimal conditions for dentin collagen cross-linking.

In summary, this study investigates the effects of cinnamaldehyde, vanillin, and dialdehyde starch on demineralized dentin, a clinically relevant substrate with structural and biochemical features distinct from reconstituted collagen. While previous work has evaluated cinnamaldehyde and dialdehyde starch on reconstituted collagen [21, 23, 41], and vanillin on denatured collagen (gelatin) [54–56], their effects on dentin have been explored now. The complex architecture of dentin collagen may influence cross-linker performance, underscoring the importance of substrate-specific evaluation. The results demonstrate the collagen-stabilizing potential of dialdehyde starch, supporting its use as a biocompatible alternative to glutaraldehyde.

## Conclusions

Dialdehyde starch did not alter the collagen triple helix, as supported by FTIR analysis, and effectively stabilized demineralized dentin against enzymatic degradation, as indicated by reduced hydroxyproline release and preserved ultrastructure. In contrast, the naturally derived aromatic aldehydes cinnamaldehyde and vanillin showed limited protective effects, likely due to their lower cross-linking capacity. Given its lower cytotoxicity, dialdehyde starch presents a biocompatible alternative to glutaraldehyde for dentin collagen stabilization; however, its performance under oral conditions warrants further investigation.

## Supplementary Information

Below is the link to the electronic supplementary material.

Supplementary Material 1 (JPG 729 KB)

## Data Availability

All data supporting the findings of this study are available within the paper.
