# Supplementary figures and images for: Preventing enzymatic degradation of demineralized dentin collagen using a natural crosslinker
Source: Clin Oral Investig. 2025 Dec 27;30(1):31. doi: 10.1007/s00784-025-06719-1 (PMC12743697; doi:10.1007/s00784-025-06719-1)

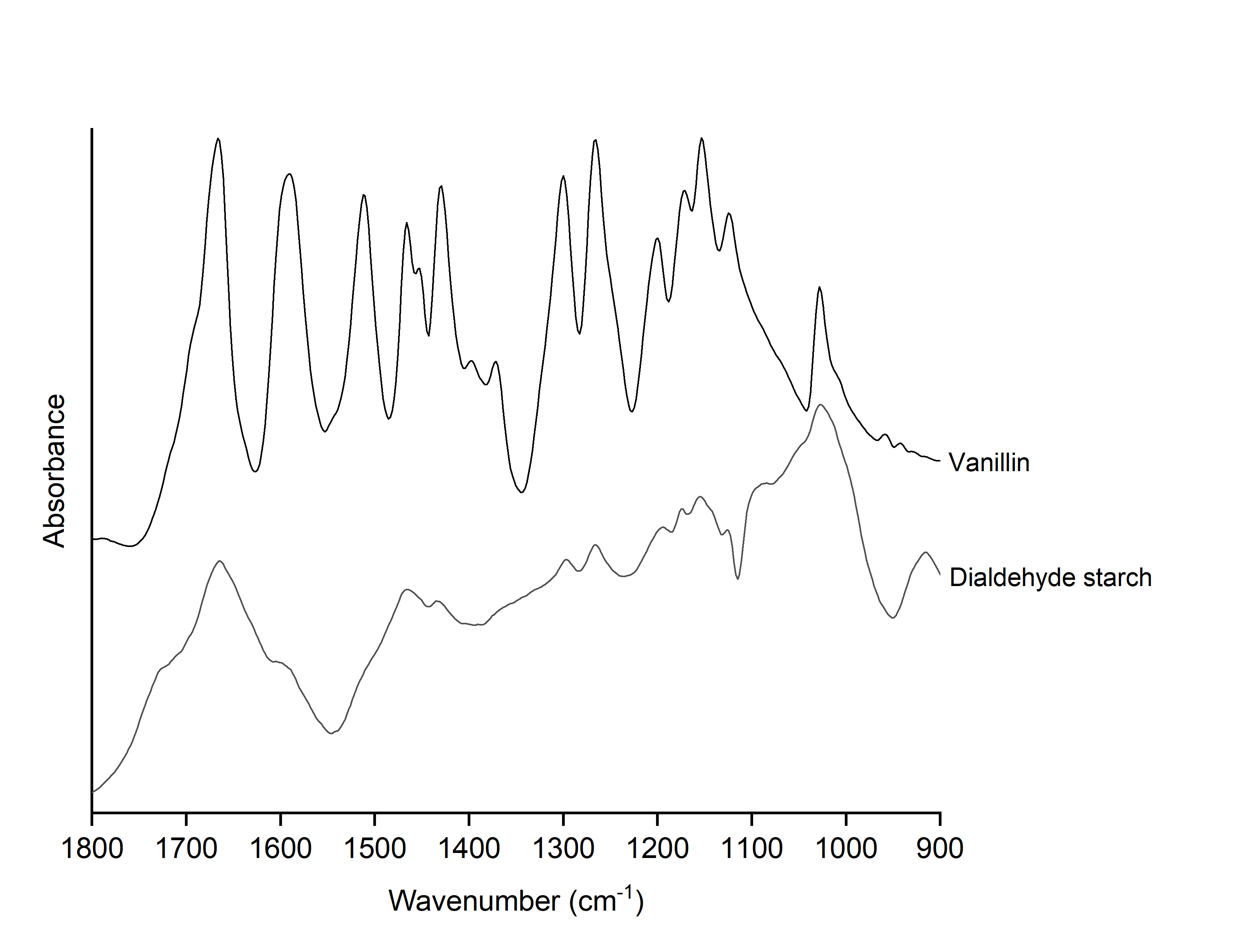

Supplement: Supplementary file 1 — Supplementary Material 1 (JPG 729 KB) [file 784_2025_6719_MOESM1_ESM.jpg]
